# Associations between nutritional and immune status and clinicopathologic factors in patients with tuberculosis: A comprehensive analysis

**DOI:** 10.3389/fcimb.2022.1013751

**Published:** 2022-11-24

**Authors:** Quan-Xian Liu, Dao-Yan Tang, Xi Xiang, Jian-Qing He

**Affiliations:** ^1^ Department of Respiratory and Critical Care Medicine, West China Hospital of Sichuan University, Chengdu, China; ^2^ Department of Tuberculosis, Affiliated Hospital of Zunyi Medical University, Zunyi, China

**Keywords:** tuberculosis, nutritional status, neutrophil/lymphocyte ratio, monocyte/lymphocyte ratio, platelet/lymphocyte ratio

## Abstract

**Objective:**

This study was designed to assess and analyze nutritional status (NS) and immune status in patients with tuberculosis.

**Methods:**

A retrospective analysis was conducted on 93 TB patients hospitalized in the tuberculosis ward of the West China Hospital of Sichuan University. Subgroup comparisons were made according to age (<65 years and ≥65 years), nutritional risk score 2002 (NRS 2002 <3 and ≥3), tuberculosis location [pulmonary tuberculosis and extrapulmonary tuberculosis (including pulmonary tuberculosis complicated with extrapulmonary tuberculosis)], and prognostic nutrition index (PNI) (<45 vs ≥45).

**Results:**

Significantly increased weight loss was associated with extrapulmonary tuberculosis (P =0.0010). Serum albumin (P =0.0214), total lymphocyte count (P = 0.0009) and PNI (P = 0.0033) were significantly decreased in older patients. Neutrophils/lymphocytes (NLR) (P =0.0002), monocytes/lymphocytes (MLR) (P < 0.0001), and platelets/lymphocytes (PLR) (P =0.0107) were higher. According to NRS 2002, higher nutritional risk was associated with lower body weight and body mass index (BMI) (P < 0.0001), higher weight loss (P = 0.0012), longer duration of hospitalization (P =0.0100), lower serum albumin level and hemoglobin concentration (P <0.01), lower creatinine level, and lower PNI (P < 0.01). 0.0001), lower total lymphocyte count (P = 0.0004), higher neutrophil and monocyte counts (P <0.05), and higher NLR (P = 0.0002), MLR (P = 0.0006), and PLR (P = 0.0156). Lower PNI was associated with lower body weight (P = 0.0001) and BMI (P =0.0074), lower total protein, albumin, and hemoglobin concentrations (P < 0.0001), and lower total lymphocyte count (P < 0.0001) and creatinine levels (P = 0.0336), higher age (P =0.0002) and NRS 2002 score, P < 0.0001), longer hos-pital stay (P = 0.0003), higher neutrophil count (P = 0.0042), and NLR, MLR, and PLR (P <0.0001) were significantly correlated. In multivariate logistic regression analysis, weight loss (OR: 0.209, 95% CI: 0.060-0.722; p =0.013) was significantly associated with higher nutritional risk (NRS 2002≥3). In multiple linear regression analysis, the NRS 2002 score was higher (B=2.018; p =0.023), and extrapulmonary tuberculosis (B=-6.205; p =0.007) was linked with a longer duration of hos-pitalization.

**Conclusions:**

Older tuberculosis patients are at nutritional risk, and older patients (≥65 years old) need to pay attention to nutritional monitoring and intervention. Older TB patients and those at risk of malnutrition have increased immune ratio and impaired immune function. Management of TB patients using basic diagnostic tools to assess nutritional and immune status and calculate PNI and immunological indexes (NLR, MLR, PLR) to improve treatment outcomes.

## Introduction

Tuberculosis (TB) is a chronic infectious disease caused by *Mycobacterium tuberculosis* (MTB). It is a major infectious disease killer worldwide and one of the thirteen leading causes of death worldwide (WHO, 2021), seriously endangering public health. There are approximately 10 million new cases of TB every year in the world, and approximately 1.5 million people die of TB every year. Malnutrition is a serious public health problem with a global prevalence of 925 million (FAO et al., 2021), and malnutrition may become more common with the coronavirus-19 pandemic and the ensuing global economic disruption (Naja and Hamadeh, 2020). Malnutrition greatly increases the risk of serious outcomes of TB, and it is strongly associated with the disease (WHO, 2019). Malnutrition may also lead to nutritionally acquired immunodeficiency syndrome (Beisel, 1996; Bhargava, 2016; FAO et al., 2019), which greatly increases the susceptibility of individuals to the progression of disease infection, thereby increasing the probability of TB developing from the incubation period to the active phase. At the same time, malnutrition also in-creases the risk of treatment failure, relapse and mortality (Sinha et al., 2019). On the other hand, anorexia, fever, and malabsorption due to metabolic disorders in TB patients can lead to or aggravate malnutrition.

Malnutrition hinders the elimination of TB globally. To implement the End TB Strategy, the World Health Organization recommends that “all people with TB need to have their nutritional status assessed and receive nutritional counseling and care as needed” (Bahmanpour and World Health Organization, 2013). It can be seen that nutritional assessment is greatly significant to the com-prehensive treatment of tuberculosis patients。It is well known that nutritional status (NS) is associated with deterioration of humoral and cellular immune responses (Jablonska et al., 2020). Correct assessment of malnutrition enables appropriate nutritional therapy to support the care of patients with TB and improve outcomes for patients with TB and malnutrition.

The current literature on comprehensive assessment of nutritional status of TB patients is limited. The aim of the study was to looks for an association between the NS and the immune system status in tuberculosis patients. Analysis of these parameters and the patients’ clinical outcome would help assess the progression of the disease.

## Materials and methods

### Inclusion and exclusion criteria

We retrospectively analyzed the medical records of 93 tuberculosis patients hospitalized in the Tuberculosis Ward of the West China Hospital, Sichuan University, from August 2021 to November 2021. Assessment of NS was performed in patients at the time of hospital admission. There were 44 males and 49 females with a mean age of 45.15 (18–85) years in the study. This study included patients aged at least 18 years with active tuberculosis. Patients older than 90 years, with a hospital stay less than 24 hours, and without complete clinical and demographic parameters were excluded.

### Patients’ general clinical characteristics

**Table S1** lists the general clinical characteristics of 93 patients. The mean body weight of the patients was 54.78 ± 10.54 (30.00-90.00) kg. The average body weight de-creased due to the disease was 1.95± 3.11 (0. 00–15. 00) kg. Nine (9.68%) patients had a weight loss of >10%. BMI was over 18.5 kg/m2 in most patients, including 56 (60.22%) patients with BMI 18.5-24.9 kg/m2 and 10 (10.75%) patients with BMI≥25 kg/m2. There were 27 (29.03%) patients with body mass index <18.5 kg/m2. Most patients (65.59%) had NRS2002 ≥3. The patient history and inpatient clinical features are shown in **Table S2**. There were 44 (47.31%) cases of pulmonary tuberculosis and 49 (52.69%) cases of extrapulmonary tuberculosis (including pulmonary tuberculosis combined with extrapulmonary tuberculosis). Seventy-five (80.65%) patients were newly treated tuberculosis patients. Fifty-one (63.75%) tuberculosis patients tested positive for the MTB nucleic acid amplification test (Xpert test or MTB DNA PCR test). Twenty-seven (29.03%) patients with malnutrition received a standard diet and oral nutritional supplements (ONS). The laboratory test values are presented in **Table S3**.

### Study design

All patients recorded their deterioration of NS (including clinical symptoms that might affect their food intake, such as loss of appetite, fever, diarrhea, constipation, etc.), weight, unintentional weight loss (WL), deterioration of food intake after disease onset and complications (type 1 and type 2 diabetes) and smoking information (including the number and duration of smoking and smoking cessation). Height and weight were measured, and laboratory blood tests were performed at hospital admission. The selected blood count parameters (hemoglobin and white blood cell (WBC), total lymphocyte, neutrophil, and monocyte counts) and biochemical parameters (serum total protein and albumin, liver and kidney parameters) were analyzed. The body mass index (BMI) and WL over the course of the disease were calculated. Patients were divided into two groups according to BMI: malnourished patients (BMI< 18.5 kg/m^2^) and well-nourished patients (BMI≥18.5 kg/m^2^), and four groups according to the World Health Organization (WHO) classification: lean patients (BMI< 18.5 kg/m^2^), normal patients (18.5≤BMI ≤ 24.9 kg/m^2^), overweight patients(25.0≤BMI≤ 29.9 kg/m^2^) and obesity patients(BMI≥30.0 kg/m^2^) (World Health Organization, 1998). Nutritional risk was assessed according to the Nutritional Risk Score 2002 (NRS 2002) of the European Society for Parenteral and Enteral Nutrition (ESPEN). The nutritional prognostic index (PNI) was calculated by the formula PNI = serum albumin (g/L) +5×total number of lymphocytes in peripheral blood (×10^9^/L) based on serum albumin concentration and total number of lymphocytes in peripheral blood (Tsukahara et al., 2017). Immunological indexes, such as the neutrophil/lymphocyte ratio (NLR), platelet/lymphocyte ratio (PLR), and monocyte/lymphocyte ratio (MLR), were calculated. Subgroup analysis was performed by age (<65 years vs. ≥ 65 years), NRS 2002 (<3 vs. ≥3), PNI (< 45 vs. ≥45), and the lesion (pulmonary tuberculosis vs. extrapulmonary tuberculosis). Clinicopathological factors and selected laboratory parameters were compared between the above subgroups. In addition, correlations between selected nutritional parameters (NRS 2002, PNI, BMI) and selected clinical factors were analyzed.

### Statistical analysis

The continuous variables are represented as the means and standard deviations. Categorical variables are expressed as numbers and percentages. Based on the type of statistical distribution, comparisons between groups were made using the parameterized Student’s T test or nonparametric Mann–Whitney U test (for continuous variables) and χ2 test or Fisher’s exact test (for categorical variables). P <0.05 was considered statistically significant. Pearson’s or Spearman’s rank correlation coefficient was used to analyze the correlation between different nutritional parameters (NRS 2002, PNI, BMI) and selected clinical factors (age, sex, tuberculosis site) and laboratory parameters. Correlation strength (as correlation coefficient) and significance (as p value) were described. The strength coefficient (r) was calculated. The strength of the correlation results was explained as follows: 0.00-0.30 (weak correlation), 0.31-0.50 (moderate correlation), 0.51-0.80 (strong correlation), and 0.81-1.00 (extremely strong correlation). Associations between nutritional parameters and clinical factors were assessed using multiple linear regression analysis. Multivariate binomial logistic regression analysis was performed to determine independent factors (NRS 2002≥3) associated with malnutrition prevalence. Relative risk was estimated using the exposure odds ratio (OR) and the corresponding 95% confidence interval (CI) from the cross-list.

## Results

### Comparison of selected clinicopathological factors and nutritional parameters depending on age

The analyzed patients were divided into a low age group (<65 years) and a high age group (≥65 years). NRS-2002 scores were significantly higher in older patients than in younger patients (3.61 ± 1.29 vs. 2.72 ± 1.25; p = 0.0082). Serum albumin (33.86 ± 6.19 g/L vs. 37.78 ± 6.41 g/L; p =0.0214) and total lymphocyte count (0.76 ± 0.32 ×10^9^/L vs. 1.24 ± 0.57 ×109/L; p = 0.0009) in older patients were significantly lower than those in the younger group. In addition, the PNI in older patients was significantly lower than that in young patients (37.68 ± 7.16 vs. 43.99 ± 8.14; p = 0.0033). NLR of older patients (7.71 ± 5.13 vs. 4.09 ± 2.99; p =0.0002), MLR (0.96 ± 0.60 vs. 0.49 ± 0.30; P < 0.0001), PLR (353.58 ± 257.80 vs. 227.55 ± 162.57; p=0.0107). The percentage of patients with diabetes mellitus (type 2) and arterial hypertension was significantly higher in older patients (33.33% vs. 4.00%; p = 0.0014 and 38.89% vs. 6.67%; p = 0.0015, respectively). All comparisons between the two age groups are shown in **Table S4**.

### Comparison of selected clinicopathological factors and nutritional parameters depending on NRS 2002 classification

There was no significant difference in the incidence of nutritional risk of pulmonary tuberculosis with extrapulmonary tuberculosis between the high NRS 2002≥3 and low NRS 2002 (NRS 2002 < 3) groups (39.34% vs. 34.38%; p = 0.3667). Patients with nutritional risk had significantly lower body weight than those without nutritional risk (52.11 ± 8.64 kg vs 61.81 ± 11.19 kg; P < 0.0001). In addition, WL was significantly higher in patients with NRS 2002≥3 than in patients with NRS 2002 < 3 (2.52 ± 3.34 kg vs. 0.81 ± 2.31 kg; p = 0.0115). The BMI of patients with NRS 2002<3 was significantly higher than that of patients with NRS 2002≥3 (22.50 ± 3.03 kg/m2 vs 19.60 ± 2.92 kg/m2; P < 0.0001); patients with nutritional risk were less likely to develop diabetes (type 2) than those without nutritional risk (12.5% vs. 8.2%; p = 0.013). Patients with nutritional risk were more likely to have frailty than those without nutritional risk (34.43% vs. 12.50%; p = 0.0276). Patients with nutritional risk had a significantly longer duration of hospitalization than those without nutritional risk (15.89 ± 13.29 days vs. 10.38 ± 5.23 days; p = 0.0268). Laboratory results showed that serum albumin levels were significantly lower in patients at nutritional risk (35.48 ± 6.30 g/L vs. 39.94 ± 5.99 g/L; p = 0.0014). There was no significant reduction in serum total protein levels in patients at nutritional risk. The hemoglobin level in the nutritional risk group was lower than that in the nonnutritional risk group (114.69 ± 20.88 g/L vs. 126.97 ± 28.11 g/L; p = 0.0192). An interesting observation was a significant decrease in creatinine in patients with nutritional risk (62.11 ± 18.85 μmol/L vs. 72.44 ± 30.67 μmol/L; p = 0.0476). The PNI of patients with high NRS 2002 was significantly lower than that of patients with low NRS 2002 (40.60 ± 7.48 vs. 46.90 ± 8.34; p = 0.0004). The NLR and MLR of patients with nutritional risk were higher than those without nutritional risk (5.67 ± 4.08 vs. 3.12 ± 2.28; p = 0.0016 and 0.68 ± 0.45 vs 0.41 ± 0.27; p = 0.0028, respectively). In addition, patients with NRS 2002 ≥ 3 had a significantly higher PLR than patients with NRS 2002 < 3 (280.06 ± 182.18 vs 198.33 ± 195.49; p= 0.0480). There were no significant differences between the NRS 2002 groups in cholesterol, high-density lipoprotein cholesterol and triglyceride levels. All laboratory results of NRS 2002 for both groups are shown in **Table S5**.

### Comparison of selected clinicopathological factors and nutritional parameters depending on the tuberculosis location

There was no significant difference in the distribution of tuberculosis location between sex and age (p > 0.05). The distribution of drug resistance and prior treatment history was similar between tuberculosis and extrapulmonary tuberculosis. However, in terms of etiological examination, the positive rate of MTB culture and MTB nucleic acid amplification test in extrapulmonary tuberculosis was significantly lower than that in pulmonary tuberculosis patients (19.51% vs 50.00%, p =0.0051) and (43.90% vs 84.62%, p =0.0002), respectively. Weight loss was significantly higher in patients with extrapulmonary location compared to tuberculosis located within the lung (2.92 ± 3.72 kg vs. 0.83 ± 1.73 kg; p = 0.0010). Additionally, the percentage of patients with WL >10% was significantly higher in patients with tuberculosis in the extrapulmonary location (14.29% vs. 2.27%; p = 0.029). Compared with extrapulmonary tuberculosis, cough and sputum production were more common (81.82% vs32.65%, p < 0.0001) and (75.00%vs24.49%, p < 0.0001) in pulmonary tuberculosis patients. The average duration of hospitalization for extrapulmonary tuberculosis patients was significantly longer than for pulmonary tuberculosis patients. In terms of laboratory examination, white blood cell count and neutrophil count in pulmonary tuberculosis patients were significantly higher than those in extrapulmonary tuberculosis patients (p < 0.05). PNI, NLR, MLR, and PLR were similar in both subgroups. All comparisons between the two general locations are presented in **Table S6**.

### Comparison of selected clinicopathological factors and nutritional parameters depending on PNI

Patients in the low PNI group (PNI<45) were generally older than those in the high PNI group (PNI≥45) (50.33 ± 19.44 years vs. 35.28 ± 16.01 years; p = 0.0003), and the NRS2002 scores of the low PNI group were higher than those of the high PNI group (3.18 ± 1.20 scores vs. 2.34 ± 1.31 scores; p = 0.0027). Compared with the high PNI group, the body weight in the low PNI group was significantly lower (53.45 ± 9.53 kg vs. 59.25 ± 11.60 kg; p = 0.0114). The serum total protein (63.28 ± 9.06 g/L vs. 71.18 ± 4.97 g/L; p< 0.0001) and albumin (33.63 ± 5.12 g/L vs. 43.47 ± 3.14 g/L; p< 0.0001) concentrations were significantly lower in the low PNI group than in the high PNI group. Patients with PNI<45 had a low total lymphocyte count (0.90 ± 0.34 ×10^9/L vs. 1.63 ± 0.58×10^9/L; p < 0.0001) and hemoglobin concentration (108.34 ± 20.70 g/L vs. 139.06 ± 16.38 g/L; p < 0.0001) compared to those with PNI≥45. The duration of hospitalization was significantly longer in patients with PNI<45 than in those with PNI≥45 (16.75 ± 12.91 days vs. 8.72 ± 4.87 days; p = 0.0741). The NLR, MLR and PLR in the low PNI group were significantly higher than those in the high PNI group (5.79 ± 4.06 vs. 2.88 ± 2.06; p =0.0003), (0.71 ± 0.45 vs. 0.35 ± 0.21; p < 0.0001) and (299.17 ± 208.06 vs. 161.90 ± 102.15; p = 0.0007). All comparisons between the low and high PNI groups are presented in **Table S7**.

### Correlations between selected nutritional parameters and clinicopathological factors

A significant positive correlation between NRS 2002 and WL (r = 0.2624; p= 0.0187), duration of hospitalization (r = 0.2659, p= 0.0100), neutrophil count (r = 0.3068; p= 0.0028), monocyte count (r = 0.2361; p= 0.0227), NLR (r = 0.3830; p= 0.0002), MLR (r = 0.3473; p= 0.0006) and PLR (r = 0.2502; p= 0.0156) was noted in our patients. NRS 2002 was negatively correlated with weight (r = -0.5687; p < 0.0001), BMI (r = -0.5862; p < 0.0001), albumin (r =-0.3921; p = 0.0001), total lymphocyte count (r =-0.3621; p = 0.0004), hemoglobin (r =-0.3272; p = 0.0014), creatinine (r =-0.2506; p = 0.0154) and PNI (r =-0.4299; p < 0.0001).

PNI was positively correlated with weight (r = 0.3845; p = 0.0001), BMI (r = 0.2761, p = 0.0074), albumin concentrations (r = 0.9571; p < 0.0001), total protein concentrations (r = 0.6320; p < 0.0001), total lymphocyte count (r = 0.7377; p < 0.0001), hemoglobin (r = 0.6365; p < 0.0001), and creatinine (r = 0.2206; p = 0.0336). and negatively correlated with age (r =-0.3814; p=0.0002), NRS 2002 (r =-0.4299; p < 0.0001), the duration of hospitalization (r =-0.3700; p = 0.0003), neutrophil count (r =-0.294; p = 0.0042), NLR (r =-0.6600; p < 0.0001), MLR (r =-0.5961; p < 0.0001), and PLR (r =-0.5014; p < 0.0001).

BMI was positively correlated with weight (r = 0.8317; p < 0.0001), albumin concentrations (r =0.2570, p =0.0129), total lymphocyte count (r =0.2206; p =0.0336), hemoglobin (r =0.2693; p =0.0090), and PNI (r =0.2761; p =0.0074). BMI was significantly negatively correlated with NRS 2002 (r =-0.5862; p < 0.0001), neutrophil count (r =-0.2512; p = 0.0152), NLR (r =-0.2976; p = 0.0038), MLR (r =-0.2532; p = 0.0143), and PLR (r =-0.2619; p = 0.0112).

All correlations are presented in **Table 1**.

**Table 1:** Correlations between NRS 2002, PNI, BMI, and selected clinicopathological and laboratory parameters.

Variable	NRS 2002		PNI		BMI	
	r	*p*	r	*p*	r	*p*
Age	0.1959	0.0599	-0.3814	**0.0002**	0.06605	0.5293
Weight	-0.5687	**< 0.0001**	0.3845	**0.0001**	0.8317	**< 0.0001**
Weight loss	0.3313	**0.0012**	-0.09969	0.3418	-0.1955	0.0604
BMI	-0.5862	**< 0.0001**	0.2761	**0.0074**		
NRS 2002			-0.4299	**< 0.0001**	-0.5862	**< 0.0001**
Duration of hospitalization	0.2659	**0.01**	-0.37	**0.0003**	-0.08516	0.417
Total protein	-0.1634	0.1176	0.632	**< 0.0001**	0.1532	0.1426
Albumin	-0.3921	**0.0001**	0.9571	**< 0.0001**	0.257	**0.0129**
Neutrophil count	0.3068	**0.0028**	-0.294	**0.0042**	-0.2512	**0.0152**
Monocyte count	0.2361	**0.0227**	-0.1652	0.1136	-0.1672	0.1092
Total lymphocyte count	-0.3621	**0.0004**	0.7377	**< 0.0001**	0.2206	**0.0336**
Hemoglobin	-0.3272	**0.0014**	0.6365	**< 0.0001**	**0.2693**	**0.009**
Creatinine	-0.2506	**0.0154**	0.2206	**0.0336**	0.1019	0.331
PNI	-0.4299	**< 0.0001**			0.2761	**0.0074**
NLR	0.383	**0.0002**	-0.66	**< 0.0001**	-0.2976	**0.0038**
MLR	0.3473	**0.0006**	-0.5961	**< 0.0001**	-0.2532	**0.0143**
PLR	0.2502	**0.0156**	-0.5014	**< 0.0001**	-0.2619	**0.0112**

NRS 2002, Nutritional Risk Score; PNI, prognostic nutritional index; BMI, body mass index; NLR, neutrophil/lymphocyte ratio; MLR, monocyte/lymphocyte ratio; PLR, platelet/lymphocyte ratio. Significant results (p < 0.05) are highlighted in bold.

### Regression analysis for association between selected nutritional parameters and clinicopathological factors

Multiple linear regression analysis showed that BMI (β=-0.495, p<0.001) and creatinine (β=-0.173, p=0.034) significantly negatively predicted NRS 2002 scores, and body weight loss (β=0.205, p =0.010) and age (β=0.195, p =0.021) significantly positively predicted NRS 2002 scores. Albumin (β=-0.114, p=0.258) and hemoglobin (β=-0.062, p =0.000) could not predict NRS 2002 scores. These variables explained 47.0% of the variation in the NRS 2002 score (**Table 2**).

**Table 2:** Association between NRS 2002 and selected clinicopathological factors and laboratory parameters in multiple regression linear analysis.

	B	β	t	p	F	Adjusted R2
BMI	-0.197	-0.495	-6.031	**＜0.001**	14.592	0.470
Weight loss	0.085	0.205	2.644	**0.01**		
Albumin	-0.023	-0.114	-1.138	0.258		
Age	0.013	0.195	2.351	**0.021**		

BMI, body mass index; SE, standard error; OR, odds ratio; 95% CI, 95% confidence intervals. Significant associations (p< 0.05) are highlighted in bold.

In multivariate logistic regression analysis, weight loss (OR: 0.209, 95% CI: 0.060-0.722; p =0.013) was significantly associated with higher nutritional risk (NRS 2002≥3) (**Table 3**).

**Table 3:** Association between NRS 2002 classification and selected clinicopathological factors in multiple binomial regression analysis.

	B	SE b	Wald Stat.	P	OR	−95% CI	+95% CI
Weight loss groups	-1.565	0.633	6.12	**0.013**	0.209	0.06	0.722
≥5% vs.<5%							
Smoking	-0.344	0.578	0.356	0.551	0.709	0.228	2.198
Tuberculosis location	0.189	0.49	0.148	0.7	1.207	0.463	3.152
Age groups≤65 vs. >65	-1.362	0.696	3.835	0.05	0.256	0.066	1.001

SE, standard error; OR, odds ratio; 95% CI, 95% confidence interval. Significant associations (p< 0.05) are highlighted in bold.

In multiple linear regression analysis, a higher NRS 2002 score was associated with a longer hospital stay (B=2.018; p =0.023), and patients with extrapulmonary tuberculosis had a longer hospital stay than patients with pulmonary tuberculosis (B=-6.205; p =0.007) (**Table 4**).

**Table 4:** Predictors for duration of hospitalization in multiple linear regression analysis.

	B	β	t	p	F	Adjusted R2
NRS 2002 groups <3 vs.≥3	2.018	0.228	2.318	**0.023**	7.516	0.124
Tuberculosis location Intrapulmonary vs. extrapulmonary	-6.205	-0.272	-2.758	**0.007**		

Significant associations (p< 0.05) are highlighted in bold.

## Discussion

This study shows some correlation between NS and clinicopathological factors. Age, BMI, WL, and low serum creatinine levels were risk factors for the NRS 2002 nutritional assessment in our patients. On the other hand, patients with NRS 2002≥3 and extrapulmonary tuberculosis had a longer hospital stay. In addition, older patients with tuberculosis and those with tuberculosis with an NRS2002 score ≥3 had increased immune ratio (NLR, MLR, and PLR). We noted a significant negative correlation between PNI and immune ratio (NLR, MLR, and PLR). Meanwhile, the total peripheral blood lymphocyte counts of the older patients with tuberculosis and those with PNI < 45 tended to be lower. Therefore, there is a relationship between various nutritional parameters and clinicopathological factors as well as nutritional status.

### Association between NRS 2002 nutritional risk assessment and NS in TB patients

The NRS 2002 is a nutritional risk screening tool that is commonly used to assess malnutrition in hospitalized patients and the potential risk of its occurrence. In this study, 61 of 93 inpatients with tuberculosis were found to have NRS 2002 ≥3, and the incidence of nutritional risk was 65.59% (61/93), suggesting that inpatients with tuberculosis had a high nutritional risk, similar to that reported in previous literature (Ali et al., 2020; Li et al., 2021). Patients with nutritional risk (NRS 2002 score ≥3) showed lower body weight and body mass index, more weight loss, and more fatigue, similar to a literature review finding that malnutrition patients had different degrees of underweight, wasting, and weakness (Verrest et al., 2021), suggesting that the NRS 2002 score could more accurately reflect the phenotype of malnutrition patients.

Our results showed that neutrophil count, monocyte count, NLR, MLR and PLR were significantly increased in patients with high NRS 2002 scores, while total lymphocyte count was decreased. In the correlation analysis, the NRS 2002 score was significantly positively correlated with neutrophil count, monocyte count, NLR, MLR, PLR, weight loss and length of hospital stay and significantly negatively correlated with total lymphocyte count, hemoglobin, albumin, creatinine, and PNI. Tuberculosis patients showed increased neutrophil counts and monocyte counts and decreased lymphocyte counts (Park et al., 2014), leading to imbalances in NLR, MLR and PLR. Studies have shown that MLR can be used as an auxiliary diagnostic indicator of active tuberculosis (La Manna et al., 2017), including extrapulmonary tuberculosis, and MLR is related to the severity of tuberculosis (Chen et al., 2022). MLR decreased after anti-tuberculosis treatment (Wang et al., 2019). In addition, after MTB infection, monocytes promote the release of inflammatory mediators, and then monocytes transform into macrophages to participate in the immune response (Refai et al., 2018). The involvement of lymphocytes in adaptive immune response and the reduction of total lymphocytes may lead to the weakening of adaptive immune response, resulting in immune dysfunction. Our results show that TB patients with nutritional impairment have impaired immune function and longer hospital stay than TB patients without nutritional risk. Patients at nutritional risk (NRS 2002 score ≥3) had lower peripheral blood monocyte count, neutrophil count, and total lymphocyte count, leading to significantly higher NLR, MLR, and PLR. These results suggest that the impaired immune function of TB patients at risk of malnutrition may be related to the reduction of total lymphocyte count. This could also explain why patients with nutritional risks are sicker. Total lymphocytes, hemoglobin, albumin and creatinine are associated with nutritional status and nutritional risk and can be used as indicators of nutritional status.

### Association between age and NS in TB patients

The NRS-2002 score of patients aged ≥ 65 years is higher than that of patients aged <65 years, suggesting a high incidence of nutritional risk in older patients, which is consistent with the results of related studies (Donini et al., 2020). The reason for the high incidence of nutritional risk in the older may be related to the aging of taste and smell, oral problems, decreased activity and other factors that lead to loss of appetite and insufficient nutritional intake in older patients (Norman et al., 2021). In addition, TB patients tend to be in a state of high catabolism and high consumption (Ren et al., 2019), resulting in increased energy consumption. Therefore, in the process of anti-tuberculosis treatment, we should pay attention to the malnutrition of older tuberculosis patients and provide nutritional intervention treatment in time.

Albumin is a structural protein produced by the liver. Insufficient intake, excessive consumption or reduced liver synthesis influenced by inflammatory mediators will lead to reduced serum albumin levels and severe inflammatory responses. Geng (Geng et al., 2015) et al. reported a higher inflammatory state and lower protein levels in patients with advanced pancreatic cancer dystrophy. The results of this study showed that the serum albumin level, total lymphocyte count and PNI of tuberculosis patients over 65 years old were lower than those under 65 years old, while NLR, MLR and PLR were higher than those under 65 years old, indicating that older tuberculosis patients were also prone to lower albumin levels and impaired immune function. In addition, the total lymphocyte count is reduced in older TB patients, and thus, the adaptive immune response is weakened, so older TB patients are often immunocompromised.

### Weight loss and BMI in patients with tuberculosis

In our study, the average weight loss of TB patients was 1.95 ± 3.11 kg, and 9.68% of TB patients lost more than 10% of their weight. Weight loss in tuberculosis patients is related to the host immune response, and cachexia appears in severe cases (Luies and du Preez, 2020). MTB infection leads to the activation of cytokines such as IL-1, IL-2, TNF-α and IFN-γ (Chang et al., 2013), which leads to a decrease in MyoD protein, a transcription factor involved in muscle development, and inhibits the activation of mRNA for myosin synthesis, leading to myosin hydrolysis (Luies and du Preez, 2020). TNF-α acts on muscle cells to promote protein loss through nuclear transcription factor κB and activate the ubiquitin–proteasome pathway to promote muscle degradation, leading to weight loss (Morley et al., 2006). Weight loss in tuberculosis patients may also be associated with decreased plasma leptin levels in the host (Skupien et al., 2018). Studies have shown that the more severe the inflammation, the lower the leptin levels, the greater the WL (van Crevel et al., 2002). Leptin levels are low in TB patients at nutritional risk (Skupien et al., 2018), so weight loss is significant in TB patients with nutritional disorders.

The basic characteristics of patients in this study showed that the average BMI was 20.57 ± 3.24 kg/m^2^, and there were 27 patients (29.03%) with BMI <18.5 kg/m^2^, suggesting that low BMI was relatively common in tuberculosis patients. Evidence suggests an increased risk of TB in people with a low BMI (Dolla et al., 2021), with an estimated hazard ratio of 12.4 (95% confidence interval [CI], 5.7 - 26.9) for TB in people with a BMI < 18.5 (Cegielski et al., 2012). In addition, low BMI is associated with adverse outcomes of TB treatment (Sinha et al., 2019). People with low BMI need a longer time for negative transformation of sputum bacteria (Putri et al., 2014), tuberculosis is prone to relapse (Khan et al., 2006), and the mortality rate caused by tuberculosis increases (Kvasnovsky et al., 2016).

### Association between tuberculosis location and NS in TB patients

Yi Li et al. found that patients with extrapulmonary tuberculosis had a higher incidence of malnutrition (Li et al., 2021). However, in our study, there was no statistically significant difference in the incidence of nutritional risk between patients with pulmonary tuberculosis and patients with extrapulmonary tuberculosis, which may be related to the small sample size of our study and the relatively mild disease of our patients with extrapulmonary tuberculosis. Compared with the extrapulmonary tuberculosis group, the number of white blood cells and neutrophils increased in the tuberculosis group, but the mechanism is unclear and needs further basic research. Studies have shown that chronic inflammation itself (Wall et al., 2013) and changes in the endocrine system caused by chronic diseases (Pende et al., 1990; Schuetz and Müller, 2006) lead to decreased appetite, catabolic activity and gastrointestinal dysfunction, re-sulting in insufficient nutritional intake and significant WL (Ellingsgaard et al., 2011). The patients with extrapulmonary tuberculosis had longer hospital stays and more obvious weight loss due to their complicated conditions, increased catabolism, and more complications.

### Serum creatinine levels in patients with tuberculosis

Serum creatinine is mainly produced by muscle metabolism and released into the blood through a nonenzymatic dehydration reaction (Wyss and Kaddurah, 2000), which indirectly reflects renal function status. Chronic inflammation can enhance oxidative stress and endothelial dysfunction associated with renal impairment (André et al., 2017). Tuberculosis, as a special chronic inflammatory disease, may have potential renal damage, so the serum creatinine level of tuberculosis patients is higher than that of healthy people (Liang et al., 2021). In contrast, our study found that TB patients at high risk of malnutrition had lower creatinine levels than TB patients at no risk of malnutrition, possibly because TB patients at risk of malnutrition have impaired muscle metabolism and reduced creatinine production due to undernutrition.

### Association between PNI and NS in TB patients

PNI is widely used to evaluate tumor prognosis and nutritional risk in surgical patients (Liu et al., 2018). Some scholars have reported that a low PNI is associated with pulmonary tuberculosis cavities and death (Nakao et al., 2019). Studies on the prognosis of pancreatic cancer have found that serum concentrations of total protein and albumin are significantly reduced in patients with low PNI (Jabłońska et al., 2021). We grouped patients according to the mean PNI value of 45 and found that the low PNI group had significantly lower serum total protein and albumin concentrations and higher NRS scores, confirming that PNI can also be used to assess nutritional risk in tuberculosis patients. Similar to tumor patients, tuberculosis patients with low PNI have low immune function, characterized by high NLR, MLR, and PLR, and PNI was negatively correlated with immune ratio (NLR, MLR, and PLR). The total lymphocyte count of tuberculosis patients in the low PNI group was reduced, which was consistent with the results of Cazares-Sosa et al. (Cázares-Sosa et al., 2019). Patients with tuberculosis have disordered immune status. The reason may be that the lymphocytes in the process of consuming and limiting Mycobacterium tuberculosis, on the one hand, through the activation of immune cells in peripheral blood to interfere with the normal lymph circulation, on the other hand, may be further changes in peripheral blood cells by changing the hematopoietic stem cell subset level total lymphocyte count (Wang et al., 2019). This study found that the PNI of patients with nutritional risk was lower than that of patients without nutritional risk, and the hospital stay was longer than that of patients without nutritional risk, which was consistent with other studies that patients with nutritional risk had lower PNI and longer hospital stay (Hiura et al., 2020).

## Strengths and limitations

In this study, the relationship between nutritional, clinical and inflammatory parameters was extensively analyzed. There are few similar studies in the global literature. Our analysis is comprehensive and involves many parameters. To our knowledge, this is the first study to examine the relationship between nutritional status and clinical and inflammatory parameters in TB patients. The study has some limitations. This was a retrospective single-center study with a relatively small number of patients. Further prospective, multicenter, large-sample studies are needed to validate our observations.

## Conclusion

Our study confirms that nutritional disorders in TB patients are associated with immune status. Older tuberculosis patients are at nutritional risk, and older patients (≥65 years old) need to pay attention to nutritional monitoring and intervention. Older TB patients and those at risk of malnutrition have increased immune ratio and impaired immune function.Management of TB patients using basic diagnostic tools to assess nutritional and immune status and calculate PNI and immune ratios (NLR, MLR, PLR) to improve treatment outcomes.

## Data availability statement

The original contributions presented in the study are included in the article/**Supplementary Material**. Further inquiries can be directed to the corresponding author.

## Ethics statement

Ethical review and approval was not required for the study on human participants in accordance with the local legislation and institutional requirements. Written informed consent for participation was not required for this study in accordance with the national legislation and the institutional requirements.

## Author contributions

All authors contributed substantially to the study design, data interpretation, and the writing of the manuscript. JH contributed to the study design. QL, DT and XX contributed to data collection, completed full text. All authors reviewed the manuscript. All authors contributed to the article and approved the submitted version.

## Funding

This work was supported by the National Natural Science Foundation of China (Grant No. 81870015) and the Science and Technology Fund Project of Guizhou Provincial Health Commission (GZWJKJ2019-1-085).

## Conflict of interest

The authors declare that the research was conducted in the absence of any commercial or financial relationships that could be construed as a potential conflict of interest.

## Publisher’s note

All claims expressed in this article are solely those of the authors and do not necessarily represent those of their affiliated organizations, or those of the publisher, the editors and the reviewers. Any product that may be evaluated in this article, or claim that may be made by its manufacturer, is not guaranteed or endorsed by the publisher.

## Supplementary material

Supplementary Table 1 The patient general clinical characteristics. BMI, body mass index; NRS 2002, Nutritional Risk Score 2002.

Supplementary Table 2 The patients clinical characteristics regarding hospitalization. AFB, acid-fast bacilli; AFB^+^, AFB smear positive; AFB^–^, AFB smear negative; NAAT, nucleic acid amplification test.

Supplementary Table 3 Laboratory results. ALT; alanine aminotransferase; AST, aspartate aminotransferase; GGT; gamma-glutamyl transpeptidase; ALP, alkaline phosphatase; PNI, prognostic nutritional index; NLR, neutrophil/lymphocyte ratio; MLR, monocyte/lymphocyte ratio; PLR, platelet/lymphocyte ratio.

Supplementary Table 4 Comparison of selected clinicopathological and nutritional parameters depending on age. NRS 2002, Nutritional Risk Score 2002; BMI, body mass index; PNI, prognostic nutritional index; NLR, neutrophil/lymphocyte ratio; MLR, monocyte/lymphocyte ratio; PLR, platelet/lymphocyte ratio. Significant results (p < 0.05) are highlighted in bold.

Supplementary Table 5 Comparison of selected clinicopathological and nutritional parameters depending on the NRS 2002 classification. AFB, acid-fast bacilli; AFB^+^, AFB smear positive; AFB^–^, AFB smear negative; TB, tuberculosis; NAAT, nucleic acid amplification test; BMI, body mass index; ALT; alanine aminotransferase; AST, aspartate aminotransferase; GGT; gamma-glutamyl transpeptidase; ALP, alkaline phosphatase; HDL-C, High-density lipoprotein cholesterol; PNI, prognostic nutritional index; NLR, neutrophil/lymphocyte ratio; MLR, monocyte/lymphocyte ratio; PLR, platelet/lymphocyte ratio. Significant results (p < 0.05) are highlighted in bold.

Supplementary Table 6 Comparison of selected clinicopathological and nutritional parameters depending on the tuberculosis location. AFB, acid-fast bacilli; AFB^–^, AFB smear negative; AFB^+^, AFB smear positive; TB, tuberculosis; NAAT, nucleic acid amplification test; BMI, body mass index; ALT; alanine aminotransferase; AST, aspartate aminotransferase; GGT; gamma-glutamyl transpeptidase; ALP, alkaline phosphatase; HDL-C, high-density lipoprotein cholesterol; PNI, prognostic nutritional index; NLR, neutrophil/lymphocyte ratio; MLR, monocyte/lymphocyte ratio; PLR, platelet/lymphocyte ratio. Significant results (p < 0.05) are highlighted in significant results (p < 0.05) are highlighted in bold.

Supplementary Table 7 Comparison of selected clinicopathological and nutritional parameters depending on PNI. PNI, prognostic nutritional index; NRS 2002, Nutritional Risk Score; BMI, body mass index; NLR, neutrophil/lymphocyte ratio; MLR, monocyte/lymphocyte ratio; PLR, platelet/lymphocyte ratio. Significant results (p < 0.05) are highlighted in bold.
